# Semen parameter variability among users of at-home sperm testing kits

**DOI:** 10.1186/s12894-022-01134-0

**Published:** 2022-11-15

**Authors:** Yana Aznavour, Felipe Navarrete, Jad Badreddine, Paul H. G. Simon, Vrushab Gowda, Stephen Rhodes, Ramy Abou Ghayda

**Affiliations:** 1 Endometrics Ltd., Claymont, DE USA; 2 Give Legacy, Inc., Boston, MA USA; 3 Urology Institute, University Hospitals Cleveland Medical Center, Cleveland, OH USA; 4 University of North Carolina Hospitals, Chapel Hill, NC USA; 5 Case Western Reserve University, Cleveland, OH USA

**Keywords:** Semen analysis, Sperm quality, Male infertility, Intra-individual variability, Inter-individual variability

## Abstract

**Background:**

Despite the generally accepted World Health Organization guidelines on semen analysis, an individual’s results can display significant variation when performed across time or in different laboratories. Semen parameters are in fact highly variable measures that can differ significantly between various analyses. Numerous researchers have discovered a wide range of semen parameters within each individual male, but only a few studies included the analysis of semen parameters variability in patients with infertility. The aim of this study was to evaluate the inter- and intra-individual variability of semen parameters in men of reproductive age with normozoospermia and those with oligozoospermia.

**Methods:**

Five hundred and thirteen who provided ≥ 2 semen samples (798 samples in total) using an at-home mail-in kit over a period of about 2 years were enrolled in the study. Semen samples collection using Give Legacy at-home mail-in semen collection kit; semen analysis at a CLIA-certified laboratory.

**Results:**

The degree of intra-subject variation across all semen parameters was lower in men with normozoospermia compared to men with oligozoospermia. Men with normozoospermia furthermore demonstrated a level of intra-subject variation that was lower than inter-subject variation across all measured parameters. No association was observed between intra-subject coefficients of variation in any of the semen parameters, including sperm concentration, sperm count, motile sperm count, total motility, progressive motility, the percentage of sperm with normal morphology, and the age, duration of abstinence, and BMI of the men.

**Conclusion:**

The results of this observational study confirm the significant variability in semen parameters in men with normozoospermia and oligozoospermia, as measured from at-home semen collection kit samples. This further underscore the importance of securing multiple samples for analysis to provide a robust assessment of male fertility.

**Supplementary Information:**

The online version contains supplementary material available at 10.1186/s12894-022-01134-0.

## Background

Semen analysis remains the single most important modality in the initial clinical evaluation of men’s reproductive health. Despite the generally accepted World Health Organization (WHO) guidelines [1] on this procedure, an individual’s results can display significant variation when performed across time or in different laboratories [2]. Semen parameters are in fact highly variable measures that can differ significantly between various analyses; for this reason, a single semen study should not be used alone in assessing fertility status [3, 4]. The American Urological Association (AUA)/the American Society for Reproductive Medicine (ASRM) guidelines on diagnosis and treatment of male infertility recommend performing at least two semen analyses, ideally spaced at least one month apart, especially in cases when the first semen analysis revealed abnormal parameters [3].

Previous studies have focused much attention on the intra-subject variation of semen parameters [5, 6]. Brooks A. Keel enrolled 74 patients with infertility and 65 healthy donors who provided 479 and 2043 semen samples, respectively, and demonstrated substantial intraindividual variability among semen parameters, particularly sperm count. Moreover, intraclass correlations demonstrated that a minimum of three semen analyses should be performed prior to making the diagnosis of infertility [7]. Similarly, Blickenstorfer et al. support the need for at least two semen analyses in male fertility assessment. The authors analyzed 5132 semen samples from 2566 men and demonstrated that only 51.2% of cases in the second analysis confirmed the initial results [8]. Mallidis et al. investigated 673 semen samples obtained from seven healthy men over a seven-year period and revealed noticeable within-subject variations in sperm concentration, ejaculate volume, and motility index [5].

The results of a semen analysis may be affected by the differences in laboratory technique and equipment, the duration of the subject’s abstinence period, seasonal changes, lifestyle factors, medications, or supplement use. Apart from the potential influence of confounding factors, the most common limitation of previously conducted studies remains the enrollment of healthy volunteers or sperm bank donors, which makes the cohort less representative. Moreover, convenience factors may limit subjects from providing more than one sample as it involves an additional interruption to their daily schedule and requires another visit to the andrology laboratory. In this study, we evaluated the inter- and intra-subject variability of semen parameters in men (with both normozoospermia and oligozoospermia) using a mail-in semen collection kit. As the fertility tech market continues to expand, providers—and patients—have increasingly turned to at-home testing solutions to evaluate sperm health. One of them provides the basis of the present study. Give Legacy, Inc. ships a transport buffer and semen collection cup to subjects’ homes, where a sample is produced, and then processes it in a fully equipped andrology laboratory. Not only does this paper represent the first such review of semen parameter variation using the at-home collection process, but it is also the most comprehensive investigation of the semen parameter variation among US men with normozoospermia and oligozoospermia. This study offers the additional benefit of drawing upon a broad cross-section of subjects who wish to test and store their sperm, allowing authors to capture a wide range of sperm health and lends further robustness to the variability studies.

## Methods

### Study population

Following IRB approval, we reviewed the de-identified records of all men who requested semen analysis at Give Legacy, Inc. laboratory more than one time during the last three years (2019–2021). Laboratory results for semen analysis and demographic data of all patients were retrieved and collected anonymously from their medical records. All subjects underwent comprehensive semen analysis within different years. Sperm concentration, total motile sperm count per ejaculate, and sperm morphology were assessed according to the World Health Organization and Kruger criteria.

### Semen samples collection and processing

All participants were clearly instructed on the sample collection procedure, which involved masturbation into sterile containers containing transportation media (mHTF Vitrolife) following at least two days of sexual abstinence (but no more than to 5–7 days). The samples were then shipped overnight to Give Legacy, Inc.’s CLIA-compliant laboratory in San Antonio, Texas for semen analysis. Ejaculates were liquified during transit, processed by centrifugation, resuspended, and washed once in mHTF (mHTF, Vitrolife) to remove seminal plasma, immature germ cells, and non-sperm cells. A portion of the purified sperm was diluted in Origio sperm wash solution (Cat#84,055,060) to assess sperm motility and morphology.

### Motility assessment

Sperm suspensions (5 µl) were loaded onto a pre-warmed chamber slide (depth, 20 μm) (Cell Vision, The Netherlands). The mixture was placed under a microscope stage at 37 °C. Sperm movement was examined using the Ceros computer-assisted semen analysis (CASA) system (Hamilton Thorne Research, Beverly, MA). One-second tracks were captured using the following settings: 60 frames per second, 60 frames acquired, minimum contrast = 80, minimum size = 3 pixels, default cell size = 6 pixels, default cell intensity = 160, slow cells counted as motile, low VAP cutoff = 10 μm/s, low VSL cutoff = 0 μm/s, minimum intensity gate = 0.18, maximum intensity gate = 1.21, minimum size gate = 0.56 pixels, maximum size gate = 2.63 pixels, minimum elongation gate = 0 pixels, and maximum elongation gate = 99 pixels. The percentage of motility was calculated by dividing non-progressive motility by progressive motility (NP/PM).

### Sperm morphology

For the Diff-Quik staining kit method, we followed the WHO manual guidelines [1]. We smeared 10 ul of semen on a slide, which was fixed by immersion in triarylmethane fixative for 15 s after complete air drying. The smears were then consecutively stained with Solution 1 (10 s), then air-dried, and stained with Solution 2 (5 s). Finally, the slides were washed in running tap water to remove the excess stain (10–15 times) [1]. The stained slides were scored using the Ceros CASA system and DIMENSIONS II Software (Hamilton Thorne, Beverly, MA) at 100-x magnification with oil immersion (Leica Microsystems) within 5 h of their preparation.

To the best of our knowledge, the latest research has demonstrated no significant difference between the CASA method and manual assessment for all sperm parameters [9]. Moreover, a recently published systematic analysis by Finelli et al. suggests that CASA systems as a valid alternative for the evaluation of semen parameters in clinical practice, especially for sperm concentration and motility [10]. This has been confirmed in the latest WHO laboratory manual for examining and processing human sperm, the sixth edition [1]. Most high-volume andrology laboratories around the nation are currently using the CASA method because of its high reproducibility and accuracy. Manual seminal studies are only used for calibration purpose or as a confirmatory study.

## Statistical analysis

Statistical analysis was performed with Prism Version 9 software for Mac OS. Means standard deviations or medians and interquartile ranges were reported according to the data distribution, and a comparison of continuous variables was performed using the Mann-Whitney U or Student t-test as required. Categorical variables were analyzed with a chi-square test. Confidence intervals (95% CI) were obtained through a Kaplan Meier analysis, and *p* < 0.05 was considered statistically significant. Statistical differences in age and semen parameters between groups of men were determined with a one-way analysis of variance.

The intra-subject coefficient of variation (CV_w_) was calculated as the standard deviation divided by the mean and multiplied by 100. The inter-subject coefficient of variation (CV_b_) was calculated as the standard deviation in the group divided by the mean in the group and multiplied by 100. The intraclass correlation coefficients (ICC) were calculated as the mean squares between subjects divided by the sum of mean squares CV_b_ and mean squares CV_w_. High ICC is seen when intra-subject variation is smaller than inter-subject variation, and conversely, the ICC values are low when intra-subject variation is larger than inter-subject. ICC values define the intra-subject’s stability of measures where elevated ICC means high stability and vice versa [7].

## Results

In this retrospective study, we investigated the inter- and intra-individual variability of semen parameters in 513 US men who requested semen analysis more than two times (2–9 times) using the Give Legacy kit between 2019 and 2021. All enrolled men completed the survey prior to semen analyses and provided information regarding their age, weight, height, and period of abstinence. To receive more comprehensive and accurate results, we decided to perform the following analyses:

I. Analysis of inter- and intra-individual variability of semen parameters in 513 men, who provided ≥ 2 semen samples and completed the survey;
II. Analysis of inter- and intra-individual variability of semen parameters in 132 men, who provided ≥ 3 semen samples and completed the survey.

In each analysis, the patients were divided into two groups according to the sperm concentration value obtained during the first semen analysis:

- Men with normozoospermia (sperm concentration ≥ 15 × 10^6^/mL),
- Men with oligozoospermia (sperm concentration < 15 × 10^6^/mL).

I. Analysis of inter- and intra-individual variability of semen parameters in 513 men, who provided ≥ 2 semen samples and completed the survey.

The mean age of men was 33.5 and 33.12 years in groups Ia and Ib respectively. The mean duration of sexual abstinence in these groups was 3.7 and 3.4 days respectively (Table 1). There were no statistically significant differences in age (*p*-value of 0.27) but in the duration of abstinence between the two mentioned groups (*p*-value of 0.011). Mean BMI in groups Ia and Ib was 25.99 and 25.89 kg/m² in groups Ia and Ib respectively (*p*-value 0.23).

**Table 1:** Characteristics of men with normozoospermia (group Ia) and oligozoospermia (group Ib)

Group	Number of patients	Number of semen samples	An average number of samples per patient	Age (years, mean)	Duration of abstinence (days, mean)	BMI (mean, kg/m²)	Current smokers (n, %)
Group Ia	339	798	2.35	33.5	3.7	25.76	26 (7.67)
Group Ib	174	426	2.44	33.12	3.4	26.10	14 (8)

For each patient we calculated mean values for the following semen parameters: sperm concentration, total sperm count, motile sperm count, total and progressive motility, and percentage of sperm with normal morphology. Then the mean values of mentioned semen parameters were calculated for each group of patients (Table 2). Men with normozoospermia (group Ia) demonstrated significantly higher values in all semen parameters when compared to patients with oligozoospermia (group Ib). As was mentioned earlier, we enrolled patients who provided more than two semen samples (2–9 times, interquartile range = 1).

**Table 2:** Mean semen parameters of men with normozoospermia (group Ia) and oligozoospermia (group Ib)

Semen parameter (mean ± SD)	Group Ia	Group Ib	*p*-value
Sperm concentration (million per mL)	55.57 ± 46.8	10.01 ± 8.3	< 0.001
Sperm count (million)	187.13 ± 119.69	42.45 ± 41.86	< 0.001
Motile sperm count (million)	62.68 ± 57.85	13.19 ± 20.99	< 0.001
Total motility (%)	30.9 ± 15.21	20.51 ± 12.13	< 0.001
Progressive motility (%)	23.06 ± 12.47	15.65 ± 9.81	< 0.001
Sperm with normal morphology (%)	7.87 ± 5.1	6.3 ± 4.12	0.003

**Fig. 1:**
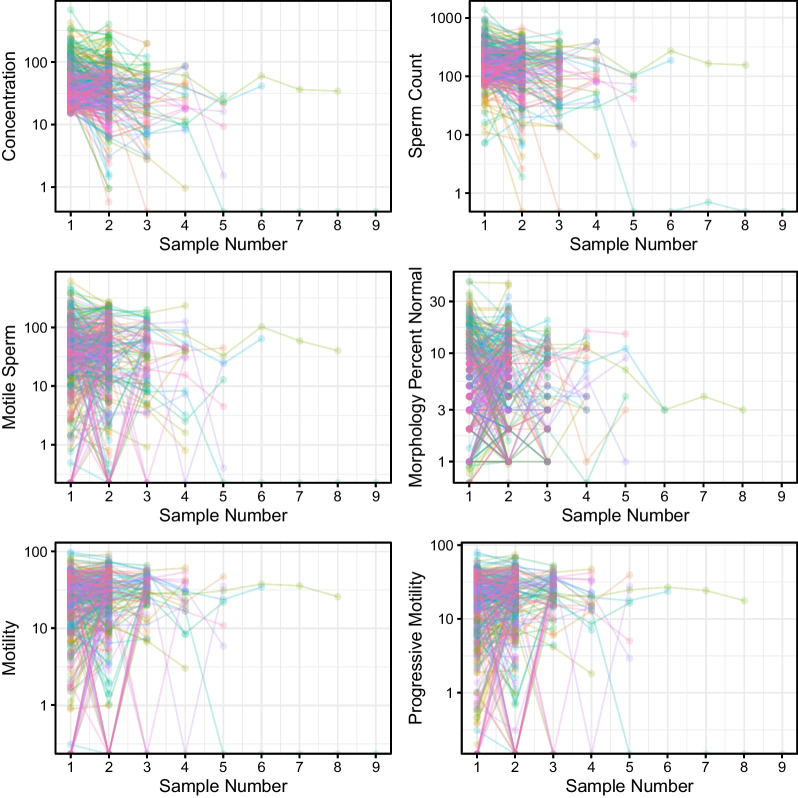
Variation in sperm concentration, sperm count, motile sperm count, total motility, progressive motility, and the percentage of sperm with normal morphology in men with normozoospermia (group Ia). Each dot represents one semen sample, each colored line represents all samples provided by each of the patients

**Fig. 2:**
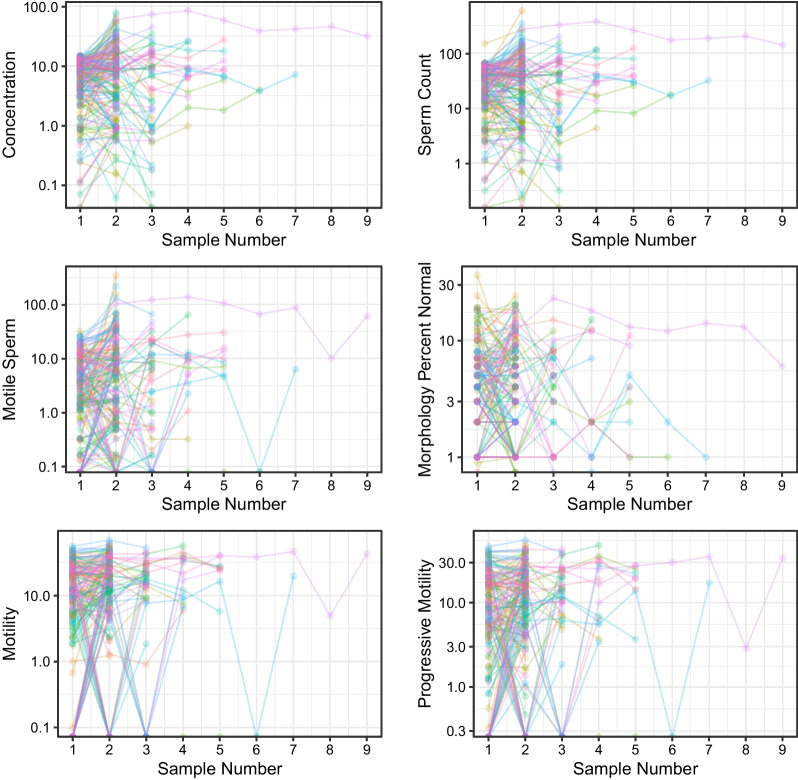
Variation in sperm concentration, sperm count, motile sperm count, total motility, progressive motility, and the percentage of sperm with normal morphology in men with oligozoospermia (group Ib). Each dot represents one semen sample, each colored line represents all samples provided by each of the patients

Variation in analyzed semen parameters in all men with normozoospermia (group Ia) and oligozoospermia (group Ib) is demonstrated on Figs. 1 and 2, respectively.

Intra-subject and inter-subject coefficients of variation were calculated for each group (Table 3). The degree of intra-subject variation in all semen parameters was lower in men from group Ia when compared to men from group Ib. In both groups, the intra-subject variation was lower than the inter-subject one for all semen parameters. The smallest degree of intra-subject variation was noted for sperm count in group Ia and total motility in group Ib, while the smallest inter-subject variation—was for total motility in both groups. The ICC values for sperm concentration, sperm count, and motile sperm count were higher than 0.75 in both groups (Table 3).

**Table 3:** Intra-subject (CV_w_ ) and inter-subject (CV_b_ ) coefficients of variation, intraclass correlation coefficients (ICC) for men from groups Ia and Ib

Semen parameter (mean)	Group Ia	Group Ib
*Sperm concentration*		
CV_w_ (%)	34.17	42.01
CV_b_ (%)	84.22	82.87
ICC	0.86	0.80
*Sperm count*		
CV_w_ (%)	29.22	40.71
CV_b_ (%)	63.96	98.62
ICC	0.83	0.85
*Motile sperm count*		
CV_w_ (%)	42.14	53.38
CV_b_ (%)	92.31	159.14
ICC	0.83	0.90
* Total motility*		
CV_w_ (%)	31.51	38.01
CV_b_ (%)	49.23	59.15
ICC	0.71	0.71
* Progressive motility*		
CV_w_ (%)	34.72	39.76
CV_b_ (%)	54.11	62.69
ICC	0.71	0.71
* Sperm with normal morphology*		
CV_w_ (%)	45.55	47.17
CV_b_ (%)	64.84	64.86
ICC	0.67	0.65

**Fig. 3:**
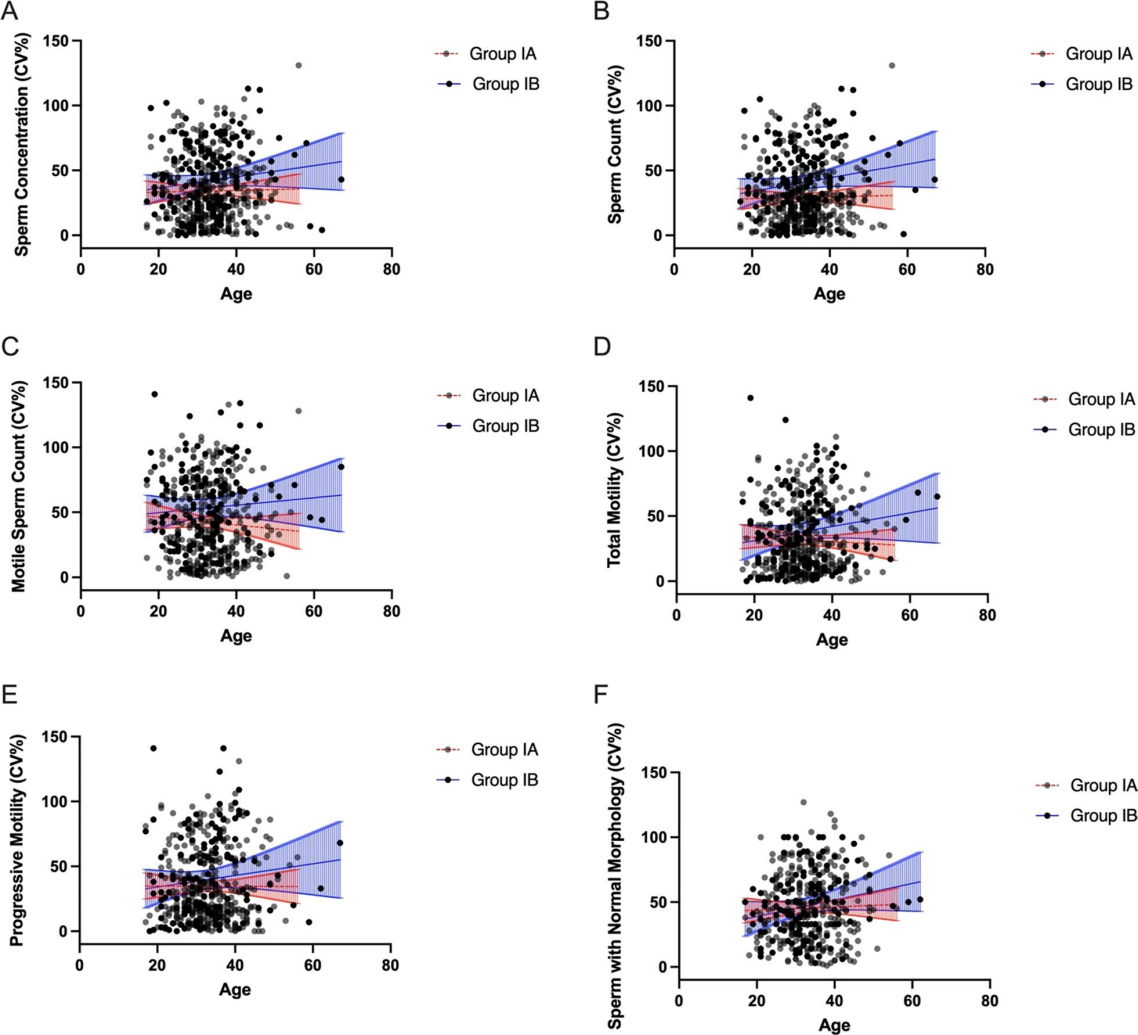
** A**–**F** Intra-subject coefficients of variation in sperm concentration (**A**), sperm count (**B**), motile sperm count (**C**), total motility (**D**), progressive motility (**E**), and the percentage of sperm with normal morphology (**F**) in groups Ia and Ib in relation to age. Lines represent the linear regression and the 95% confidence interval (blue lines—group Ia, red lines—group Ib)

**Fig. 4:**
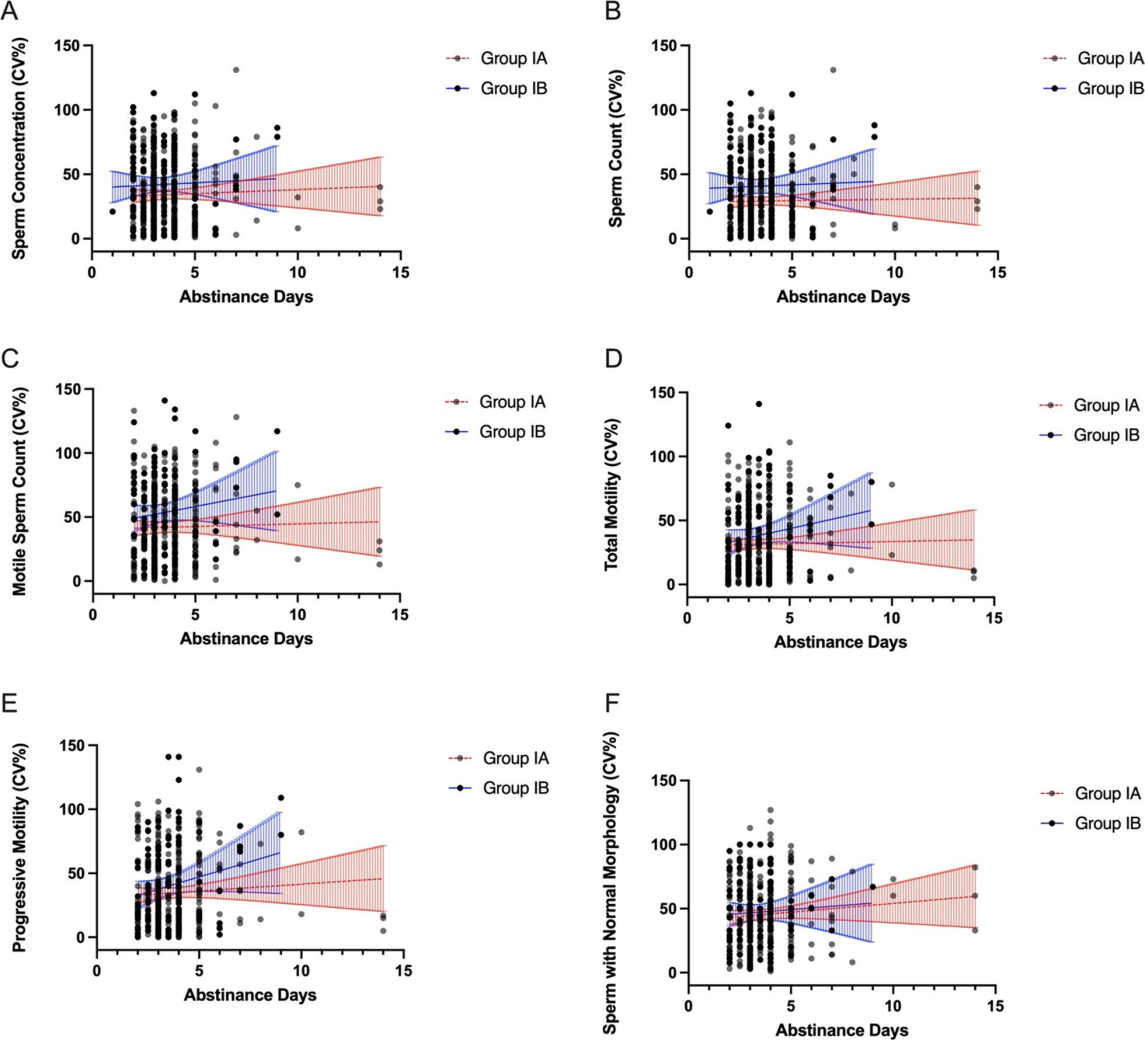
** A**–**F** Intra-subject coefficients of variation in sperm concentration (**A**), sperm count (**B**), motile sperm count (**C**), total motility (**D**), progressive motility (**E**), and the percentage of sperm with normal morphology (**F**) in groups Ia and Ib in relation to the duration of abstinence. Lines represent the linear regression and the 95% confidence interval (blue lines—group Ia, red lines—group Ib)

**Fig. 5:**
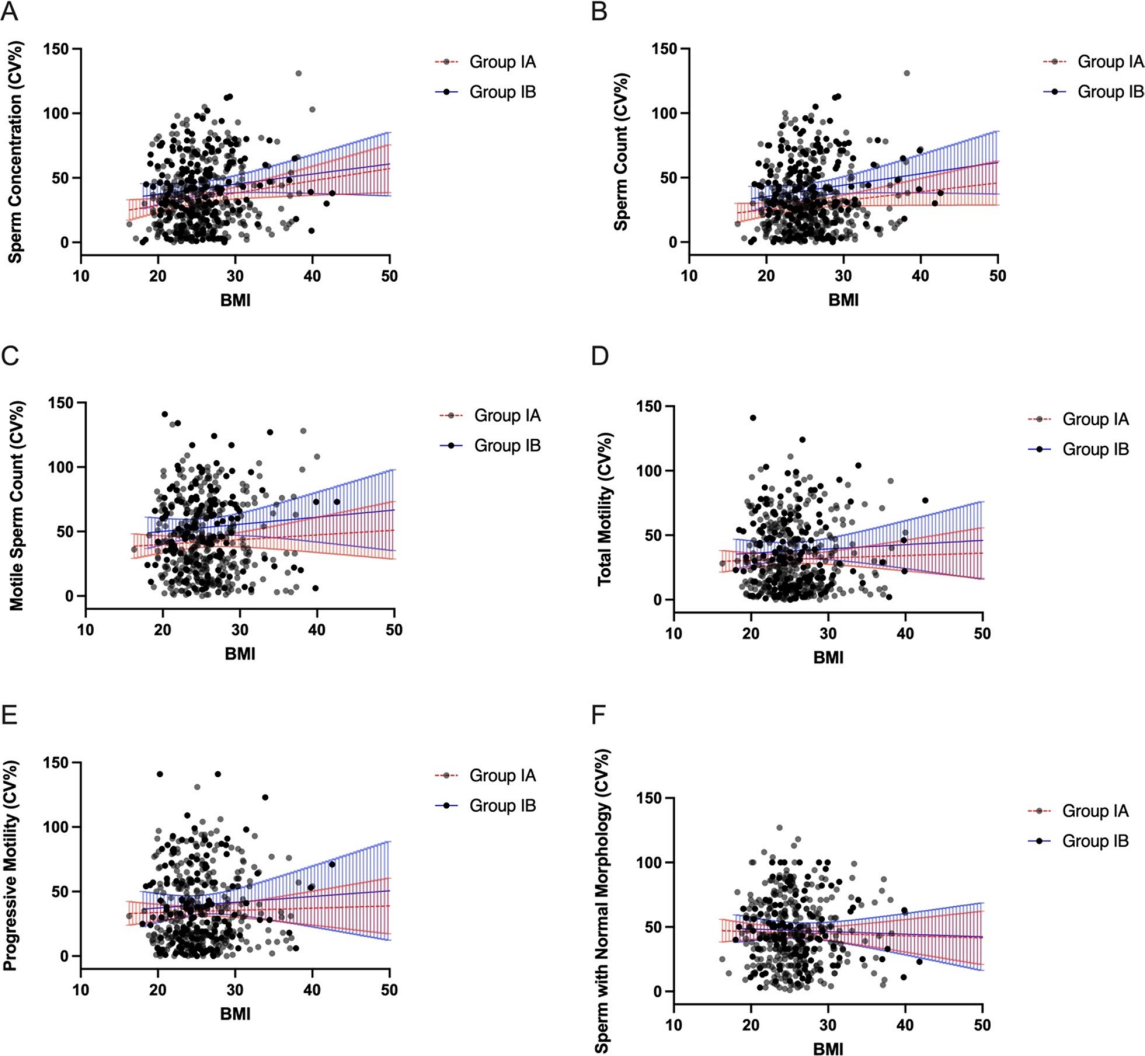
** A**–**F** Intra-subject coefficients of variation in sperm concentration (**A**), sperm count (**B**), motile sperm count (**C**), total motility (**D**), progressive motility (**E**), and the percentage of sperm with normal morphology (**F**) in groups Ia and Ib in relation to BMI. Lines represent the linear regression and the 95% confidence interval (blue lines—group Ia, red lines—group Ib)

To determine whether age, the duration of abstinence or BMI affects the intra-subject coefficients of variation in each of the semen parameters, we adjusted obtained CV_w_ values for mentioned factors. We haven’t observed an association between intra-subject coefficients of variation in any of the semen parameters, including sperm concentration, sperm count, motile sperm count, total motility, progressive motility, the percentage of sperm with normal morphology, and the age, duration of abstinence, and BMI of the men (Figs. 3A–F, 4A–F, 5A–F).

II. Analysis of inter- and intra-individual variability of semen parameters in 132 men, who provided ≥ 3 semen samples and completed the survey.

132 men out of 513 provided ≥ 3 semen samples and we decided to analyze whether the larger number of samples affect obtained results. The mean age of men was 33.59 and 33.86 years, the mean duration of sexual abstinence in these groups was 3.63 and 3.5 days, mean BMI was 25.99 and 25.89 kg/m² in groups IIa and IIb respectively (Table 4). There were no statistically significant differences in both parameters (*p*-value of 0.42 and 0.29 respectively).

**Table 4:** Characteristics of men with normozoospermia (group IIa) and oligozoospermia (group IIb) who provided ≥ 3 semen samples and completed the survey

Group	Number of patients	Number of semen samples	Age (years, mean)	Duration of abstinence (days, mean)	BMI (mean, kg/m²)	Current smokers (n, %)
Group IIa	87	291	33.59	3.63	25.99	8 (9.19)
Group IIb	45	168	33.86	3.5	25.89	1 (2.22)

For each patient of two new groups, we again calculated mean values for sperm concentration, total sperm count, motile sperm count, total and progressive motility, and percentage of sperm with normal morphology. Then the mean values of listed semen parameters were calculated for each group (Additional file 1: Table S1). Men with normozoospermia (group IIa) again demonstrated significantly higher values in all semen parameters when compared to patients with oligozoospermia (group IIb).

We divided this cohort into two groups based on the age, duration of abstinence, and BMI, then the mean values of semen parameters were measured for each new group (Additional file 2: Table S2). When the cohort was divided by age, the mean total motility, mean progressive motility, mean percentage of sperm with normal morphology, and mean motile sperm count were significantly higher in all semen analysis in younger men (< 35 years old). When cohort were divided by the duration of abstinence or BMI, no statistically significant differences were found between groups (Additional file 3: Table S3).

Intra-subjects and inter-subjects’ coefficients of variation were again calculated for each group (Additional file 4: Table S4). The degree of within-subject variation in all semen parameters was lower in men from group IIa when compared to men from group IIb. The smallest degree of within-subject variation was noted for sperm count in both groups, while the smallest between-subject variation—was for total motility in both groups.

**Fig. 6:**
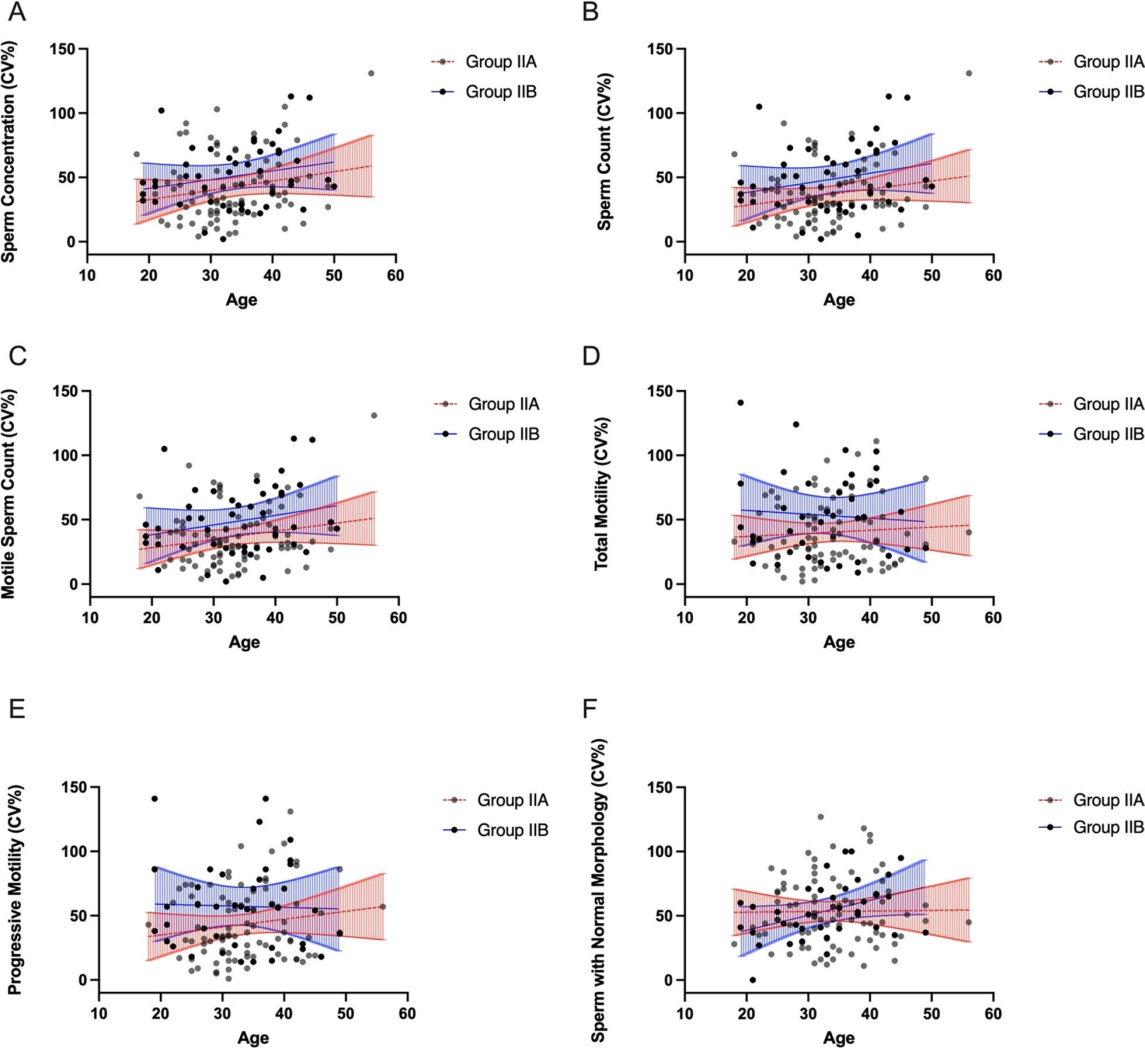
** A**–**F** Intra-subject coefficients of variation in sperm concentration (**A**), sperm count (**B**), motile sperm count (**C**), total motility (**D**), progressive motility (**E**), and the percentage of sperm with normal morphology (**F**) in groups IIa and IIb in relation to age. Lines represent the linear regression and the 95% confidence interval (blue lines—group IIa, red lines—group IIb)

**Fig. 7:**
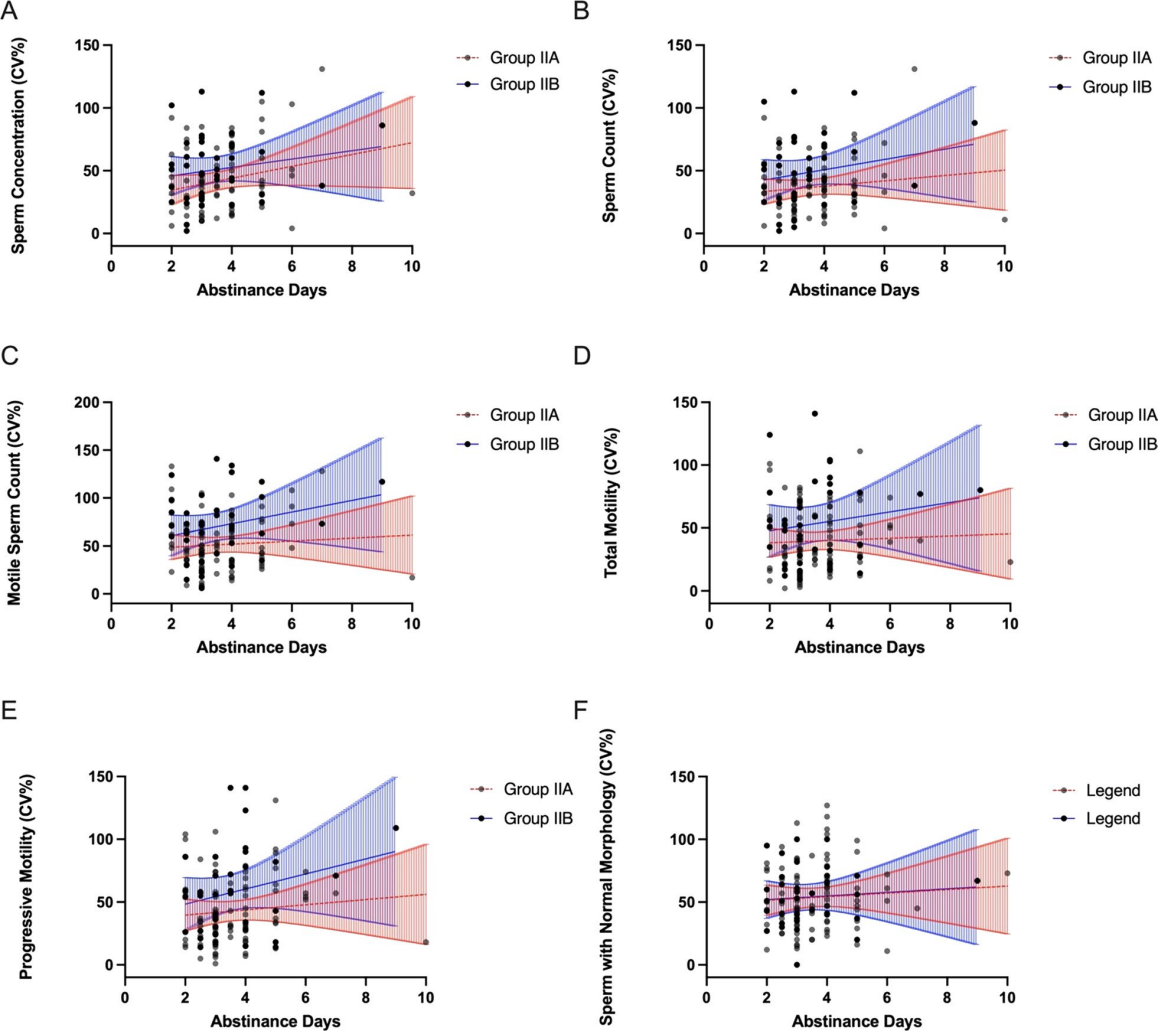
** A**–**F** Intra-subject coefficients of variation in sperm concentration (**A**), sperm count (**B**), motile sperm count (**C**), total motility (**D**), progressive motility (**E**), and the percentage of sperm with normal morphology (**F**) in groups IIa and IIb in relation to the duration of abstinence. Lines represent the linear regression and the 95% confidence interval (blue lines—group IIa, red lines—group IIb)

**Fig. 8:**
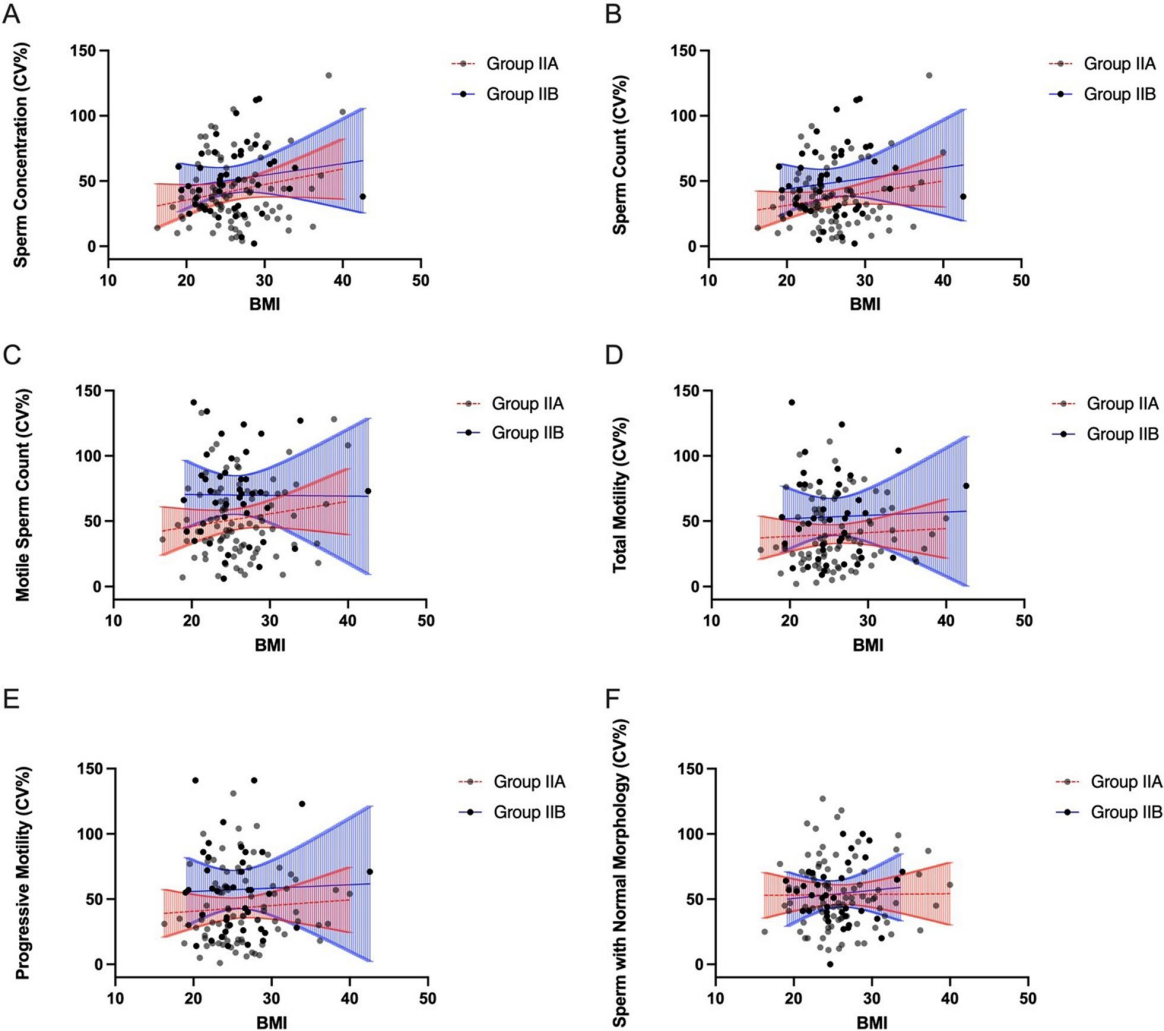
** A**–**F** Intra-subject coefficients of variation in sperm concentration (**A**), sperm count (**B**), motile sperm count (**C**), total motility (**D**), progressive motility (**E**), and the percentage of sperm with normal morphology (**F**) in groups IIa and IIb in relation to BMI. Lines represent the linear regression and the 95% confidence interval (blue lines—group IIa, red lines—group IIb)

We also adjusted obtained CV_w_ values for the age, the duration of abstinence and BMI and again have not observed an association between within-subject coefficients of variation in any of the semen parameters, including sperm concentration, sperm count, motile sperm count, total motility, progressive motility, the percentage of sperm with normal morphology, and the age, duration of abstinence, and BMI of the men of groups IIa and IIb (Figs. 6A–F, 7A–F, 8A–F).

## Discussion

Multiple semen samples should be analyzed during the clinical assessment of men’s fertility status to make a more accurate diagnosis and to avoid unnecessary treatment. Differences in semen parameters across different analyses from samples provided by the same men may indicate the extensive impact of seasonal changes, lifestyle factors, level of physical activity, and medication or supplement use.

Numerous researchers have discovered a wide range of semen parameters within each individual male. Nevertheless, only a few studies included the analysis of semen parameters variability in patients with infertility. Keel enrolled 139 men who provided 2522 semen samples and revealed large intra-subject variation in semen parameters, especially sperm count, in both patients and healthy donors’ cohorts. The author noted that at least three semen samples should be analyzed during a man’s fertility status assessment [7]. Francavilla et al. conducted a similar study involving 436 men with infertility who provided five semen samples each (2180 samples in total) and revealed high within-subject coefficients of variation for semen volume, sperm concentration, and forward motility [11]. A modified study design was adopted by Christian Fuglesang S. Jensen et al., who compared the results of semen analyses from 11 patients and 17 healthy donors in both university-based and private laboratories at 1 year intervals. Researchers haven’t revealed any intra-subject significant differences in semen volume, total motile sperm count, and sperm concentration. At the same time, sperm motility was significantly higher when analyzed at the private laboratories while the percentage of sperm with normal morphology was significantly higher when analyzed at the university-based laboratory [2].

In this study, we investigated the inter- and intra-individual variability of semen parameters in both normozoospermic and oligozoospermic US men and performed two similar analyses on men who provided ≥ 2 and ≥ 3 semen samples, using an at-home sperm collection kit. Men were assigned to either group (a) (men with normozoospermia) or (b) (men with oligozoospermia) based on sperm concentration value in the first semen analysis result. Each additional semen analysis performed for these men was added to the matched patient profile. The groups of men were homogenous in terms of age and the duration of sexual abstinence. Semen analyses were performed in the same laboratory, allowing accurate comparative analysis of semen parameters of a large group of men.

The within-subject variation (CV_w_) represents the variability of each value for individual patients, while the between-subject variation (CV_b_), by contrast, demonstrates the ​​difference between all patients in the chosen cohort. In this study, the degree of within-subject variation in all semen parameters was lower in men with normozoospermia than in men with oligozoospermia in both cohorts of men who provided ≥ 2 and ≥ 3 semen samples. This observation suggests that one semen analysis results are more representative of men with normozoospermia than of men with oligozoospermia, which agree with the previously published data by Keel.

In a cohort of men who provided ≥ 2 semen samples, the within-subject variation was lower than between-subject one for all parameters in men with normozoospermia and for all parameters except sperm concentration in men with oligozoospermia. In a cohort of men who provided ≥ 3 semen samples, the within-subject variability was significantly higher, which potentially suggests that analyzing 2 semen samples may not be sufficient for characterizing men’s fertility status. Interestingly, inter-subject variability was significantly lower in both men with normozoospermia and oligozoospermia who provided ≥ 3 semen samples and even lower than intra-subject variation in most parameters. The CV_b_ was only lower than CV_w_ in sperm concentration among men with normozoospermia, and in motile sperm count in men with oligozoospermia. Nevertheless, the results might be attributed to the lower number of patients in the second cohort.

The smallest degree of intra-subject variation among men who provided ≥ 2 semen samples was noted for sperm count in the group with normozoospermia and for total motility in the group with oligozoospermia, while in the cohort of men who provided ≥ 3 semen samples, the smallest degree of within-subject variation was noted for sperm count in both groups. As for the between-subject variation, the smallest degree was observed for total motility for all groups of both cohorts.

A test’s reliability measures how easily the results can be replicated and demonstrates both the degree of correlation and the degree of concordance between several sets of measurements [12]. The ICC values for sperm concentration, sperm count, and motile sperm count were higher than 0.75 in both men with normozoospermia and oligozoospermia, who provided ≥ 2 semen samples, but notably lower in the cohort of men who provided ≥ 3 semen samples. Further studies should be conducted on a larger number of men who provided ≥ 4–5 semen samples to investigate the change in CV_w_, CV_b_, and ICC levels with the increased number of collected semen samples.

When the cohort was divided by age, the mean total motility, mean progressive motility, mean percentage of sperm with normal morphology, and mean motile sperm count was significantly higher in all semen analyses in younger men (< 35 years old). Nevertheless, we have not observed an association between within-subject coefficients of variation across any of the semen parameters, including sperm concentration, sperm count, motile sperm count, total motility, progressive motility, the percentage of sperm with normal morphology, and the age, duration of abstinence, and BMI of the men. Thus, our results suggest that the CV_w_ values reflect the biological variation in semen parameters assumed in earlier studies [13].

We have shown that there can be significant variability between semen samples, especially among oligospermic patients. These results will open the way for further larger studies assessing if more than two semen analyses are needed in specific sub-groups of infertile men better to evaluate a true reflection of their seminal parameters.

## Strengths and limitations of the study

We believe that the major strengths of the present research are represented by the largest cohort of the US male population, the fact that all semen analysis was performed in the same laboratory, and that random control lab performance assessments were completed to ensure accurate data during collection. Moreover, the evaluation of all semen analyses was conducted with a CASA system to facilitate objective, reproducible, and large-scale analysis, which was calibrated with manual standard semen analysis according to WHO references. For the morphological assessment, staining and evaluation processes were centralized, and thus the results are not likely to be affected by any technical issues. Additionally, this study is the first major analysis of sperm parameter variability from an at-home semen collection kit in the literature. This method of semen collection is growing increasingly popular as the fertility tech space matures, and more individuals opt to provide a semen sample from the privacy and convenience of their own home.

By the same token, the main limitation of this study derives from the fact that semen samples were collected at home, mixed with transportation media, and then shipped overnight to the lab, so data cannot be strictly compared with other studies using traditional andrology assessment, particularly in terms of sperm morphology. Moreover, only those men who ordered an at-home mail-in semen collection kit were enrolled in the study, therefore results may not be generalized to the entire population writ large. In addition, we divided men into two groups based on the sperm concentration value in the first semen analysis result as we lack information regarding underlying fertility status.

## Conclusion

The study results confirm the already existing and published data regarding the variability of semen parameters. The novelty of this study is that it was done using at-home, mail-in semen kits. This confirms, similar to other reports, that at-home, mail-in semen kits provide a satisfactory screening tool that would pave the way for further guideline-guided testing for male factor infertility. Semen analysis done at a physical Andrology laboratory is still the gold standard, yielding the most accurate seminal studies. However, given the dynamic shifts in the field, and the changes the Covid-19 pandemic created, at home, mail-in kits might have a role to play. Using these kits can generate momentum in the right direction of testing. It can provide affordable access to initial fertility care, opening subsequent opportunities and discussions for further reproductive health assessment.

We believe that the presented data contribute new insights to the literature on the inter- and intra-individual variability of semen parameters and support the necessity of a comprehensive fertility status assessment involving multiple analyses.

## Supplementary information

**Additional file 1: Table S1.** Mean semen parameters of men with normozoospermia (group IIa) and oligozoospermia (group IIb).

**Additional file 2: Table S2.** Characteristics of men who provided ≥ 3 semen samples and completed the survey.

**Additional file 3: Table S3.** Comparative analysis of mean semen parameters in patient cohort divided by age, duration of abstinence, and body mass index.

**Additional file 4: Table S4.** Intra-subject (CV_w_) and inter-subject (CV_b_) coefficients of variation, intraclass correlation coefficients (ICC) for men from groups IIa and IIb.

## Data Availability

The datasets generated and/or analyzed during the current study are not publicly available due to their containing information that could compromise the privacy of research participants but are available from the corresponding author upon reasonable request.
