# Supplementary material for: Semen parameter variability among users of at-home sperm testing kits
Source: BMC Urol. 2022 Nov 15;22:184. doi: 10.1186/s12894-022-01134-0 (PMC9665028; doi:10.1186/s12894-022-01134-0)
Supplement: Supplementary file 1 — Additional file 1: Table S1. Mean semen parameters of men with normozoospermia (group IIa) and oligozoospermia (group IIb). [file 12894_2022_1134_MOESM1_ESM.docx]

Supplemental Table 1

**Mean semen parameters of men with normozoospermia (group IIa) and oligozoospermia (group IIb).**

| **Semen parameter (mean±SD)** | **Group IIa** | **Group IIb** | **p-value** |
| --- | --- | --- | --- |
| Sperm concentration  (million per mL) | 50.09±25.43 | 9.64±4.24 | <0.001 |
| Sperm count (million) | 169.68±52.63 | 40.94±19.18 | <0.001 |
| Motile sperm count  (million) | 57.45±26.07 | 10.91±10.0 | <0.001 |
| Total motility (%) | 30.76±6.5 | 18.3±4.94 | <0.001 |
| Progressive motility (%) | 22.87±5.11 | 13.41±3.94 | <0.001 |
| Sperm with normal morphology (%) | 7.6±2.89 | 5.5±1.7 | 0.004 |
