# Supplementary material for: Semen parameter variability among users of at-home sperm testing kits
Source: BMC Urol. 2022 Nov 15;22:184. doi: 10.1186/s12894-022-01134-0 (PMC9665028; doi:10.1186/s12894-022-01134-0)
Supplement: Supplementary file 2 — Additional file 2: Table S2. Characteristics of men who provided ≥ 3 semen samples and completed the survey. [file 12894_2022_1134_MOESM2_ESM.docx]

Supplemental Table 2

**Characteristics of men who provided ≥3 semen samples and completed the survey.**

| **Semen parameter (mean) /the number of semen sample** | **Age** | | **Duration of abstinence** | | **BMI** | |
| --- | --- | --- | --- | --- | --- | --- |
|  | <35 (n=75) | ≥35 (n=57) | ≤3  (n=68) | >3  (n=64) | ≤24.9  (n=63) | ≥25.0  (n=69) |
| Sperm concentration  (million per mL)  #1  #2  #3 | 48.11  42.32  33.40 | 40.57  30.94  21.19 | 36.77  31.45  27.21 | 53.46  43.74  29.11 | 41.03  32.99  25.53 | 48.36  41.43  30.50 |
| Sperm count (million)  #1  #2  #3 | 151.07  140.32  126.70 | 137.53  105.1  90.58 | 133.66  117.77  108.53 | 157.51  132.91  113.84 | 139.34  112.65  106.98 | 150.59  136.49  114.87 |
| Motile sperm count  (million)  #1  #2  #3 | 58.2  45.8  46.51 | 33.94  29.63  28.87 | 44.99  36.61  40.04 | 50.62  41.16  37.68 | 47.88  36.01  38.54 | 47.58  41.38  39.22 |
| Total motility (%)  #1  #2  #3 | 29.64  27.1  30.44 | 21.56  19.96  23.96 | 27.07  24.48  29.64 | 25.17  23.51  25.52 | 27.77  24.99  29.33 | 24.66  23.12  26.1 |
| Progressive motility (%)  #1  #2  #3 | 21.77  19.66  22.82 | 15.82  15.16  17.66 | 20.1  18.68  22.35 | 18.28  16.69  18.66 | 20.43  18.36  21.75 | 18.08  17.13  19.48 |
| Sperm with normal morphology (%)  #1  #2  #3 | 9.37  7.36  7.15 | 6.66  5.26  4.67 | 8.04  6.98  6.87 | 8.53  5.89  5.24 | 8.4  6.56  6.75 | 8.14  6.39  5.55 |
