# Supplementary material for: Semen parameter variability among users of at-home sperm testing kits
Source: BMC Urol. 2022 Nov 15;22:184. doi: 10.1186/s12894-022-01134-0 (PMC9665028; doi:10.1186/s12894-022-01134-0)
Supplement: Supplementary file 3 — Additional file 3: Table S3. Comparative analysis of mean semen parameters in patient cohort divided by age, duration of abstinence, and body mass index. [file 12894_2022_1134_MOESM3_ESM.docx]

|  | **Age** | | |  | **Duration of abstinence** | | |  | **BMI** | | |
| --- | --- | --- | --- | --- | --- | --- | --- | --- | --- | --- | --- |
|  | age<35 | age≥35 | p-value |  | duration of abstinence ≤3 | duration of abstinence >3 | p-value |  | BMI≤24.9 | BMI≥25.0 | p-value |
| N | 75 | 57 |  |  | 68 | 64 |  |  | 63 | 69 |  |
| concentration-1 | 48.11951035 | 40.57403165 | 0.2425217626 |  | 36.76819306 | 53.46009301 | 0.06474196043 |  | 41.02952806 | 48.35975091 | 0.2482572 |
| concentration-2 | 42.32108535 | 30.93782172 | 0.08913802002 |  | 31.44750565 | 43.73604462 | 0.08130392145 |  | 32.99762169 | 41.43024744 | 0.1625754243 |
| concentration-3 | 33.40240542 | 21.19390244 | 0.0086144701 |  | 27.21101704 | 29.10755761 | 0.365488401 |  | 25.53269227 | 30.50251062 | 0.1764331821 |
|  |  |  |  |  |  |  |  |  |  |  |  |
| sperm count-1 | 151.0693915 | 137.5311249 | 0.3084598328 |  | 133.6585599 | 157.5108813 | 0.1837403998 |  | 139.3413622 | 150.5938066 | 0.3314990709 |
| sperm count-2 | 140.3236174 | 105.0929171 | 0.0375150867 |  | 117.7734078 | 132.9058727 | 0.2275934383 |  | 112.6477897 | 136.4892294 | 0.1133251001 |
| sperm count-3 | 126.7046911 | 90.58168378 | 0.02286980047 |  | 108.534062 | 113.8389311 | 0.3879181688 |  | 106.9838612 | 114.869921 | 0.3333844433 |
|  |  |  |  |  |  |  |  |  |  |  |  |
| total motility-1 | 30.03796661 | 23.19046152 | 0.02128556355 |  | 28.31796681 | 25.98767979 | 0.2496077742 |  | 28.22572893 | 26.18321224 | 0.2757689947 |
| total motility-2 | 27.45838547 | 21.46571103 | 0.03860684764 |  | 25.6143377 | 24.26889127 | 0.3466821223 |  | 25.3966471 | 24.53863214 | 0.4006314901 |
| total motility-3 | 30.85152229 | 25.77414323 | 0.03656482685 |  | 31.00787487 | 26.3472641 | 0.04892174177 |  | 29.80563217 | 27.70912378 | 0.2303999282 |
|  |  |  |  |  |  |  |  |  |  |  |  |
| prog.motility-1 | 22.06260265 | 17.02007782 | 0.03324830684 |  | 20.99067376 | 18.87585365 | 0.2241442544 |  | 20.75619711 | 19.19711538 | 0.2873580291 |
| prog.motility-2 | 19.92805826 | 16.30395683 | 0.08508093228 |  | 19.54281527 | 17.23391985 | 0.1903979071 |  | 18.6525681 | 18.1896431 | 0.4304548526 |
| prog.motility-3 | 23.13420644 | 18.99890801 | 0.03465606435 |  | 23.38739115 | 19.27145525 | 0.03431054431 |  | 22.10539259 | 20.70632586 | 0.2700193959 |
|  |  |  |  |  |  |  |  |  |  |  |  |
| norm.morhpol.-1 | 9.372541156 | 6.734693878 | 0.02411189758 |  | 8.037006237 | 8.627896474 | 0.3400396054 |  | 8.403423424 | 8.225646758 | 0.4491693488 |
| norm.morhpol.-2 | 7.359749455 | 5.343129788 | 0.01923005345 |  | 6.984848485 | 5.978357065 | 0.15230515 |  | 6.55865078 | 6.473604826 | 0.4660172489 |
| norm.morhpol.-3 | 7.146245059 | 4.840277778 | 0.002601648651 |  | 6.866003788 | 5.396226415 | 0.04879092025 |  | 6.75 | 5.695479384 | 0.1157087549 |
|  |  |  |  |  |  |  |  |  |  |  |  |
| motile sperm count-1 | 58.98778609 | 36.5056441 | 0.03030267528 |  | 47.07679763 | 52.25650744 | 0.3456940159 |  | 48.65552711 | 50.51157888 | 0.4425144667 |
| motile sperm count-2 | 46.4216325 | 31.86375753 | 0.06178098579 |  | 38.30112086 | 42.49043708 | 0.3304290094 |  | 36.5913297 | 43.92796173 | 0.218477844 |
| motile sperm count-3 | 47.14427612 | 31.05283299 | 0.02044286427 |  | 41.88788983 | 38.89941521 | 0.357559071 |  | 39.16117252 | 41.63821362 | 0.3806453843 |
