# Supplementary material for: Semen parameter variability among users of at-home sperm testing kits
Source: BMC Urol. 2022 Nov 15;22:184. doi: 10.1186/s12894-022-01134-0 (PMC9665028; doi:10.1186/s12894-022-01134-0)
Supplement: Supplementary file 4 — Additional file 4: Table S4. Intra-subject (CVw) and inter-subject (CVb) coefficients of variation, intraclass correlation coefficients (ICC) for men from groups IIa and IIb. [file 12894_2022_1134_MOESM4_ESM.docx]

Supplemental Table 4

**Intra-subject (CV_w_) and inter-subject (CV_b_) coefficients of variation, intraclass correlation coefficients (ICC) for men from groups IIa and IIb.**

| **Semen parameter (mean)** | **Group IIa** | **Group IIb** |
| --- | --- | --- |
| **Sperm concentration**  CV_w_ (%)  CV_b_ (%)  ICC | 42.26  50.77  0.59 | 50.90  44.01  0.43 |
| **Sperm count**  CV_w_ (%)  CV_b_ (%)  ICC | 36.82  31.02  0.42 | 48.70  46.87  0.48 |
| **Motile sperm count**  CV_w_ (%)  CV_b_ (%)  ICC | 51.43  45.39  0.44 | 70.03  91.74  0.63 |
| **Total motility**  CV_w_ (%)  CV_b_ (%)  ICC | 39.75  21.16  0.22 | 53.39  27.00  0.20 |
| **Progressive motility**  CV_w_ (%)  CV_b_ (%)  ICC | 43.01  22.35  0.21 | 57.37  29.38  0.21 |
| **Sperm with normal morphology**  CV_w_ (%)  CV_b_ (%)  ICC | 53.83  38.09  0.33 | 53.99  31.17  0.25 |
